# Obstacles to Birth Surname Retention Upon Marriage: How Do Hostile Sexism and System Justification Predict Support for Marital Surname Change Among Women?

**DOI:** 10.3389/fpsyg.2021.702553

**Published:** 2021-10-04

**Authors:** Maria Chayinska, Özden Melis Uluğ, Nevin Solak, Betül Kanık, Burcu Çuvaş

**Affiliations:** ^1^School of Psychology, Pontificia Universidad Católica de Chile, Santiago, Chile; ^2^School of Psychology, University of Sussex, Brighton, United Kingdom; ^3^Department of Psychological and Brain Sciences, University of Massachusetts Amherst, Amherst, MA, United States; ^4^Department of Psychology, TED University, Ankara, Turkey; ^5^Department of Psychology, Çanakkale Onsekiz Mart University, Çanakkale, Turkey

**Keywords:** marital surname change, system justification, hostile sexism, benevolent sexism, gender inequality

## Abstract

Despite the ongoing shift in societal norms and gender-discriminatory practices toward more equality, many heterosexual women worldwide, including in many Western societies, choose to replace their birth surname with the family name of their spouse upon marriage. Previous research has demonstrated that the adherence to sexist ideologies (i.e., a system of discriminatory gender-based beliefs) among women is associated with their greater endorsement of practices and policies that maintain gender inequality. By integrating the ideas from the system justification theory and the ambivalent sexism theory, we proposed that the more women adhere to hostile and benevolent sexist beliefs, the more likely they would be to justify existing gender relations in society, which in turn, would positively predict their support for traditional, husband-centered marital surname change. We further argued that hostile (as compared to benevolent) sexism could act as a particularly strong direct predictor of the support for marital surname change among women. We tested these possibilities across three cross-sectional studies conducted among women in Turkey (Study 1, *N*=118, self-identified feminist women; Study 2, *N*=131, female students) and the United States (Study 3, *N*=140, female students). Results of Studies 1 and 3 revealed that higher adherence to hostile (but not benevolent) sexism was associated with higher support for marital surname change indirectly through higher gender-based system justification. In Study 2, the hypothesized full mediation was not observed. Consistent with our predictions, in all three studies, hostile (but not benevolent) sexism was found to be a direct positive predictor of the support for marital surname change among women. We discuss the role of dominant ideologies surrounding marriage and inegalitarian naming conventions in different cultures as obstacles to women’s birth surname retention upon marriage.

## Introduction

“It is a very odd and radical idea indeed that.

a woman would nominally disappear.

just because she got married.”

Ellen Goodman, a Pulitzer Prize-winning U.S. columnist,

The Name of the Game, Boston Globe 30 (September 24, 1974).

Social scientists have documented substantial progress toward gender egalitarianism in the last half-century, sometimes referred to as a “gender revolution” (e.g., England et al., 2020). It can be noticed in a radical shift in the public support for practices and legal standards aimed to promote and secure greater equality of rights and opportunities between men and women (e.g., Scarborough et al., 2019; England et al., 2020). Despite the ongoing progress toward gender egalitarianism, one form of a gendered practice – women’s adoption of their husband’s surname upon marriage – remains resistant to change in many cultures and countries.^1^ In fact, according to the nationally representative opinion poll conducted in the United States a few years ago (e.g., Bame, 2017), 57% of United States adults thought that it is ideal for a woman to take their husband’s surname. Although, cultural surname practices vary worldwide, this kind of marriage-related gendered naming practice may arguably seem somewhat obsolete in the 21st century. Noteworthy, the legal doctrine law of coverture, which implied that a wife’s legal identity was subsumed under that of her husband upon marriage, was abolished almost 2 centuries ago (e.g., Kopelman et al., 2009; MacEacheron, 2016).

Prevailing support for marital surname change among heterosexual couples presents an important social issue as it manifests that there remains a social facet of status inequality in marriage, wherein (traditionally) women are expected to change their legal identity in a way men are not. Surprisingly, research that has provided insight into the social-psychological processes that underlie inegalitarian naming conventions is rare (for an exception, see, e.g., Pilcher, 2017; Stoiko and Strough, 2017). Previous research on marital surname change has approached this phenomenon from an individual perspective, which focused mainly on the role of women’s personal motives in their marital surname choice (e.g., Scheuble et al., 2012; MacEacheron, 2016; Stoiko and Strough, 2017; Taniguchi and Kaufman, 2020). While, we acknowledge the importance of understanding individual-level motives regarding naming choices, in the current paper, we argue that decisions made by individuals in relation to their surnames upon marriage can be embedded in and become a consequence of a broader social system as well.

Central to our idea is the view that male-oriented naming practices are part of a broader constellation of dominant ideologies about gender and marriage, and these ideologies are often taken for granted (Emens, 2007; Scheuble et al., 2012). Therefore, prevailing support for marital surname change can be considered a group- and system-based phenomenon, in which marital naming convictions are produced and reinforced congruent with advantaged group’s interests (i.e., men). In the present article, we raised the important question of whether the adherence to *sexist ideologies* (i.e., a system of discriminatory gender-based beliefs) among women would be associated with their endorsement of marital surname change and whether this link would be mediated by gender-based system justification. We tackled this question by drawing on the Ambivalent Sexism Theory (Glick and Fiske, 1996) and the System Justification Theory (Jost and Kay, 2005). These theories provide explanations about how sexist ideology is used to rationalize current social and political arrangements as fair and legitimate, especially among historically disadvantaged social groups. In particular, we aim to investigate the extent to which women’s adherence to *hostile sexism*, the ideology that resentfully preserves male-dominated gender relations, compared to *benevolent sexism* (i.e., a set of favorable group ascriptions that justify the current gender status quo), predicts women’s support for marital surname change directly and indirectly through gender-based system justification. We test these possibilities among self-identified women in *WEIRD* (i.e., Western, Educated, Industrialized, Rich, and Democratic; see Henrich et al., 2010) and non-WEIRD^2^ contexts: Turkey and the United States A scholarly understanding of the processes underlying women’s support for traditional (husband-centered) naming practices can help make significant progress toward understanding the obstacles of achieving gender equality.

### Ambivalent Sexism and Support for Marital Surname Change

The proponents of the Ambivalent Sexism Theory (Glick and Fiske, 1996, 2001) have argued that sexism is reflecting a profound ambivalence rather than a constant antipathy toward women (or men). The essence of sexism generally lies in an uncritical acceptance of male supremacy and female subordination. According to this theory, sexist beliefs may be organized along two different yet complementary dimensions. The first dimension reflects hostile sexism, which involves strong feelings of antipathy or animosity toward the opposite gender (Glick et al., 2004; Sibley et al., 2007; Laurin et al., 2011). Individuals adhering to such aggressive sexist beliefs tend to perceive individuals from the other gender as competing over power and dominance. The second dimension, benevolent sexism, comprises subjectively positive yet patronizing beliefs about women in their respective restricted roles. Individuals adhering to benevolent sexist beliefs typically depict women as fragile and vulnerable creatures deserving men’s protection and guidance (Glick et al., 2001). Benevolent sexism, thus, entails an affective expression of male dominance.

Both forms of ambivalent sexism have been considered as a *system-justifying ideology*, that is, the ideology that justifies, naturalizes, and perpetuates gender inequality in society (Jost and Kay, 2005; see also Sibley et al., 2007). Previous research has suggested that widely and persistently held sexist beliefs propel women to justify the dominant patriarchal ideology surrounding the marriage (e.g., Chen et al., 2009; Day et al., 2011), endorse social norms that are likely to reinforce and perpetuate male privilege in society (e.g., Glick et al., 2004; Sibley et al., 2007; Laurin et al., 2011), and support the policies aimed to restrict women’s autonomy (e.g., Petterson and Sutton, 2018; Salmen and Dhont, 2020).

While the association between ambivalent sexism and support for male-centerd marital surname change has not been systematically examined quantitatively, a few studies conducted with female college students in the context of the United States have shown that their plans to adopt their husbands’ surname upon marriage were associated with their higher conformity to patriarchal norms (e.g., Scheuble et al., 2012; Stoiko and Strough, 2017). A qualitative study conducted with professional feminist middle-class heterosexual women in the United Kingdom has suggested that they viewed the practice of marital surnaming as built into the dominant ideologies of institutionalized sexism (e.g., Mills, 2003). Research on marital surname change has also examined women’s rationales for their decision not to retain their maiden name upon marriage (e.g., Scheuble et al., 2012; MacEacheron, 2016), drawing comparisons between the choices of feminist and non-feminist women (e.g., Stoiko and Strough, 2017) as well as approaching the complex surname choices made by same-sex couples (e.g., Underwood and Robnett, 2019).

However, the claim, we wish to make here is that women’s personal choices in relation to marital surname change rarely happen in a vacuum. When a disproportionate number of women worldwide manifest the willingness to undergo a major and visible change in their legal identity by adopting their husbands’ surname upon marriage, it can reasonably be considered a group phenomenon worth scrutiny from a social-psychological perspective. Some studies have documented that sexist ideology exerts a great influence on the endorsement of patriarchal norms in a marriage that promote and protect male dominance in a heterosexual family (e.g., Chen et al., 2009; Stoiko and Strough, 2017). A handful of experimental research has shown that both benevolent sexism and hostile sexism predict individuals’ support for traditional gender roles, thus often showing their complementary role in promoting gender inequalities (e.g., Barreto and Ellemers, 2005; Barreto et al., 2010; Brownhalls et al., 2021). Nevertheless, there are reasonable grounds to suggest that higher hostile sexism may exert a particularly strong influence on women’s support for inegalitarian naming practices compared to higher benevolent sexism. This is because women’s resistance to or the lack of endorsement of this convention at the societal level may be perceived by others as a counter-stereotypical, agentic, and even system-challenging behavior that threatens the entrenched traditional gender roles (e.g., Kahn et al., 2021). As previous research has revealed, hostile (as compared to benevolent) sexism is the ideology that motivates individuals to engage in a number of different strategies aimed to preserve the stability and reaffirm the legitimacy of the gender status quo in different life domains (e.g., Connor and Fiske, 2019). So while benevolent sexism robustly predicts positive attitudes toward women who sustain traditional gender roles in the institution of marriage (e.g., Chen et al., 2009; Szastok et al., 2019), hostile sexism as the ideology reinforces idealized notions of traditional (male-dominated) gendered division and penalizes those who challenge it through agentic behavior (e.g., Connor and Fiske, 2019). Based on the previous research, we, therefore, argue that hostile sexism can *directly* predict women’s support for the traditional (husband-centered) naming practice to a greater extent than benevolent sexism.

### Ambivalent Sexism, System Justification, and Support for Marital Surname Change

Endorsement of patriarchal practices such as husband-centered marital surname change can also be affected by the extent to which women justify the existing arrangements. The current study sought to address the link between ambivalent sexism and support for marital surname change through the mediating role of gender-based system justification. System justification theory (Jost and Kay, 2005; see also Jost, 2020) offers a cognitive-motivational analysis of why and how individuals justify a social, political, and economic status quo. According to system justification theory, not only advantaged groups but also disadvantaged groups perpetuate the existing social arrangements (Jost et al., 2003; but see Owuamalam et al., 2018 for a critique of this idea). This happens because the status quo serves the disadvantage to satisfy their epistemic (e.g., to reduce uncertainty), existential (e.g., to reduce distress and threat), and relational (e.g., to connect with mainstream society) needs. In doing so, people can satisfy their inner psychological needs for stability, predictability, and control, thus avoiding the rocky path of challenging the existing societal arrangements (e.g., Hennes et al., 2012; Jost, 2020).

Both forms of ambivalent sexism – hostile and benevolent sexism – have been associated with higher levels of system justification (e.g., Glick and Fiske, 2001; Jost and Kay, 2005; Sibley et al., 2007; Brandt, 2011). In particular, studies have found that hostile sexism, a rawer form of gender-related ideology, was transversally and causally related to gender-based system justification among members of the disadvantaged group (e.g., women; see Sibley et al., 2007; Laurin et al., 2011) and predicted individuals’ support for policies aimed to restrict women’s autonomy and legitimize men’s dominance in decision-making processes (e.g., Petterson and Sutton, 2018; Salmen and Dhont, 2020). Likewise, benevolent sexism plays a complementary role in predicting individuals’ support for restrictive policies and traditional gender roles (e.g., Chen et al., 2009; Barreto et al., 2010; Kahn et al., 2021; see also Glick and Fiske, 2001). However, while hostile sexism penalizes women for gender role deviance, benevolent sexism is more likely to have a pacifying effect on women decreasing their motivation to demand social change (e.g., Becker and Wright, 2011).

In gender settings, previous research conducted with women as members of the historically-disadvantaged group has revealed that women are more likely than men to rationalize the persistent gender gap in high-status jobs and earnings (e.g., O’Brien et al., 2012). The observed pattern of behaviors is arguably consistent with the idea that women’s adherence to sexist ideologies is also reflected in their tendency to justify the current gender division, which, in turn, produces their support for policies and practices aimed to preserve the entrenched male-dominated status quo. Extending this line of research, we argue that both hostile and benevolent sexism can be associated with greater gender-based system justification and thus act as the indirect predictors of women’s support for the marital surname change.

## The Current Research

**Figure 1:**
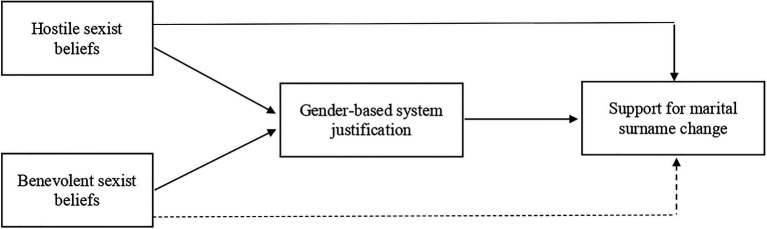
A theoretical model depicting the indirect and direct relationship between hostile and benevolent sexism on support for marital surname change through gender-based system justification.

Marital surname change represents a particularly fertile issue in which to explore whether the adherence to sexist ideologies among women predicts their support for the gendered practice that implies the replacement of women’s previous surname with the family name of their spouse upon marriage. Consistent with our theoretical backdrop, we hypothesize that women’s adherence to both forms of sexist beliefs will predict their higher tendency to justify existing gender relations in society, which in turn, will positively predict their support for marital surname change. We further argue that hostile (as compared to benevolent) sexism can act as a particularly strong direct predictor of the support for the traditional (husband-centered) naming practice among heterosexual women thus manifesting its predictive power *above and beyond* benevolent sexism. We examined these direct and indirect associations across three correlational studies, controlling for women’s political orientation because previous research has linked right-leaning political ideology to the endorsement of hostile sexism (e.g., Sibley et al., 2007). The hypothesized theoretical model is depicted in Figure 1.

We test the applicability of our theoretical model among women in Turkey and the United States The two countries represent so-called Western (the United States) and non-Western (Turkey) societies that differ substantially in the objective scores of gender inequalities and sexism such that these scores are higher in developing countries as compared to more established democracies (Gender Equality Index, 2020). Despite these objective differences, we argue that marital surname change – as an entrenched and prevailing feature of heterosexual marriage in many cultures worldwide – holds promise as one avenue into capturing the impact of social-psychological mechanisms pertaining to the support for male dominance and the patriarchal family system among women as members of a historically disadvantaged group. This is because the predictions along with the system justification theory and the ambivalent sexism theory were shown to be sustained in a number of cross-national studies, thus revealing their potential applicability in both individualist and collectivist cultures (e.g., Glick et al., 2001, 2004; Brandt, 2011). Therefore, we expect to find similar findings across the two contexts: Turkey and the United States

Finally, in the present research, we test the applicability of our model among two subpopulations of women: self-identified feminist women in Turkey (Study 1) and female university students in Turkey and the United States (Studies 2 and 3, respectively). In Study 1, we chose to focus on self-identified feminist women because previous research has found that even women who generally endorse egalitarian values tend to endorse surname change upon marriage (e.g., Mills, 2003; Stoiko and Strough, 2017). In Studies 2 and 3, we chose to focus on the female student subpopulations because young women in emerging adulthood (18–25years of age) are likely to be particularly impressionable to the processes of gender socialization by which they are taught how to behave in accordance with their assigned gender (Nielson et al., 2020). Besides, some earlier research, conducted a decade ago, has reported that support for marital surname change was also observed among highly-educated heterosexual women and female college students in Western cultures such as the United States (e.g., Scheuble et al., 2012). So, if there is a general trend for women to support marital surname change upon marriage, it has to be tested within diverse female subpopulations. Therefore, we investigate whether there are significant associations between women’s adherence to hostile sexist beliefs and higher support for marital surname change through gender-based system justification, with a particular focus on the subpopulations of feminists and female students, who might be seen as the frontrunners of social change in society.

## Study 1: Feminist Women in Turkey

In Turkey, the gendered practice of changing a woman’s surname upon marriage has been one of the most debated legal issues with respect to achieving more gender egalitarianism (e.g., Inal, 2020; Kartal, 2020). According to Article 187 of the Turkish Civil Code of 1926, a married woman is required to adopt her husband’s last name upon marriage. The article was amended in 1997 to allow women to keep their maiden surname before the surname of their husbands. This rule has not only been in conflict with the Turkish Constitution but also with the international agreements on gender equality (i.e., Convention on the Elimination of All Forms of Discrimination Against Women) to which Turkey became a party (e.g., Inal, 2020; Kartal, 2020). The only legal possibility for women in Turkey to retain their family surname upon marriage without adding that of their husband is to file a lawsuit to use this right (Inal, 2020; Kartal, 2020). The last years have indeed witnessed numerous high-profile cases of such lawsuits to the national and international courts (e.g., Uluğ, 2015).

In the recent decade, there have been both progress and significant backlash in the centuries-old struggle of feminist women in Turkey for gender equality. Many women’s hard-won rights have become a target of conservative religious groups and right-wing populist parties in this country (Kabasakal-Arat, 2020). Simultaneously, patriarchal attitudes have gained increased influence in Turkey as a result of the Islamic resurgence over the last generation (Engin and Pals, 2018; Kavas and Thornton, 2019). As of 2020, the Global Gender Gap Index has ranked Turkey as having the 130th largest gender gap of 153 countries (World Economic Forum, 2020). It is within this context Study 1 was conducted.

### Method

#### Participants and Procedure

Data were collected between May 24 and June 27, 2017. We distributed the link to the survey on various Facebook groups of female associations in Turkey concerned with women’s rights and gender equality in this Middle-Eastern country. The common requirements for participants in this study included identifying as a female, being 18years or older, and categorizing themselves as feminists. We reached the participants through snowball convenience sampling. The study was advertised as a research project seeking to understand attitudes toward various social issues among feminist women in Turkey. Written informed consent to participate in this online study was provided by all participants. Respondents were informed that there was no monetary compensation for their participation. Two hundred seventy-six volunteers entered the survey, 157 withdrew from participation without completing the survey. One hundred eighteen participants self-identified as women whereas one was a man. A male participant was excluded from the study as this person did not match the advertised inclusion criteria (i.e., being a female). The final sample consisted of 118 self-identified feminist women from Turkey. Participants’ age ranged from 21 to 65 (*M*=33.02; *SD*=9.53). Participants were highly educated (47.9% indicated they completed a Bachelor’s degree, and 32.5 earned an MSc degree). When asked regarding their marital status, 41.5% indicated they were single without a prior experience of marriage, 50% reported they were single and divorced, and 8.5% were married. We received IRB approval for this research from the University of Massachusetts Amherst.

Sensitivity analysis conducted using G*Power (Faul et al., 2009) indicated that the final sample size (*N*=118) was sufficient for detecting a small effect in a regression analysis (multiple regression: *R*^2^ deviant from zero; power=0.80; *α*=0.05; Cohen’s *f*^2^=0.10).

#### Measures

Except for the socio-demographic variables mentioned above, all items were presented on seven-point response scales (1=*completely disagree*, 7=*completely agree*). The scales were presented in random order.^3^

##### Hostile and Benevolent Sexism

To measure both forms of sexism, we used the shortened scales adapted from the Ambivalent Sexism Inventory^4^ (ASI; Glick and Fiske, 1996; see Sakallı-Uğurlu, 2002 for the adaptation of ASI to Turkish). We assessed hostile sexism with six items with the following items: “*Once a woman gets a man to commit to her, she usually tries to put him on a tight leash,” “Women seek to gain power by getting control over men*,” “*When women lose to men in a fair competition, they typically complain about being discriminated against*,” “*Women exaggerate problems they have at work*,” “*Many women are actually seeking special favours, such as hiring policies that favor them over men, under the guise of asking for ‘equality’*” and “*Men should be willing to sacrifice their own well-being in order to provide financially for the women in their lives*”^5^ (Cronbach’s *α*=0.85). Benevolent sexism was assessed with two items adapted from the ASI. These items were: “*Women, compared to men, tend to have a superior moral sensibility*” and “*Women, as compared to men, tend to have a more refined sense of culture and good taste*” (*r*=0.64, *p*<0.001). To evaluate the viability of the two-factor structure of the Ambivalent Sexism scale, we conducted Exploratory Factor Analysis (EFA) with Varimax rotation. Results revealed that the six items measuring hostile sexism loaded on one component (46.67%) and the two items measuring benevolent sexism loaded on another factor (18.22%), which together explained 64.89% of the total variance (KMO=0.815; *p*<0.001).

##### Gender-Based System Justification

We assessed gender-specific system justification with three items^6^ adapted from Jost and Kay (2005) and adjusted to the marriage context. These items were: “*In general, relations between men and women are fair*,” “*Generally speaking, women and men have equal rights in recruitment and promotion*,” and “*Generally speaking, the relationships between men and women in marriage are just and equal*” (Cronbach’s *α*=0.84).

##### Support for Marital Surname Change

We created five items to measure support for marital surname change. These items were: “*For a healthy marriage, a woman should not use her maiden name, but use only her husband’s last name*,” “*When women get married, one of the most important indicators of being a real family is women not using their maiden name but using only their husbands’ last names,*” “*A woman who loves and respects her partner should not use her own surname after marriage, but only her husbands’ surname after marriage,*” “*When women get married, a woman not using her maiden name indicates that she loves her husband*” and “*I think a woman who is not using her maiden name, but using only her husband’s last name is pure and honest*.” Results of PCA revealed that these items loaded on one factor, which explained 67% of the variance (KMO=0.855, *p*<0.001). The scale showed adequate internal consistency (Cronbach’s *α*=0.87).

##### Political Orientation

As ambivalent sexism was systematically shown to be correlated with right-wing ideology (Sibley et al., 2007; Petterson and Sutton, 2018) as well as support for power-related ideology in marriage (e.g., Chen et al., 2009), we used political orientation as a control variable. We asked participants to indicate their political orientation on a scale ranging from 1 (*extreme left*) to 9 (*extreme right*).

### Results and Discussion

#### Preliminary Analysis

Means, SDs, and correlations between the variables are presented in Table 1. The mean scores of hostile sexism, gender-based system justification, and the support for marital surname change were rather low among feminist participants. The analysis of descriptive statistics revealed that participants scored relatively high on benevolent sexism. Pearson correlation analyses were computed to analyze bivariate associations between the study constructs. Participants’ adherence to hostile sexist beliefs was found to be positively correlated with greater gender-based system justification, right-leaning political orientation, greater support for marital surname change. Benevolent sexism beliefs were found to be significantly associated only with hostile sexism beliefs, while their association with all the other study variables was found to be non-significant. Last, greater endorsement of gender-based system justification was significantly associated with greater support for marital surname change.

**Table 1:** Means, SDs, and correlations among key variables (Study 1).

Variables	*M (SD)*	1	2	3	4	5
1. Benevolent sexism	3.52 (1.84)	–				
2. Hostile sexism	1.85 (1.07)	0.31^***^	–			
3. Gender-based system justification	1.48 (0.88)	0.12	0.64^***^	–		
4. Political orientation	2.27 (1.20)	−0.05	0.30^***^	0.29^***^	–	
5. Support for marital surname change	1.44 (0.81)	0.13	0.58^***^	0.76^***^	0.24^***^	–

*** *p*<0.001.

#### Mediation Analyses

**Figure 2:**
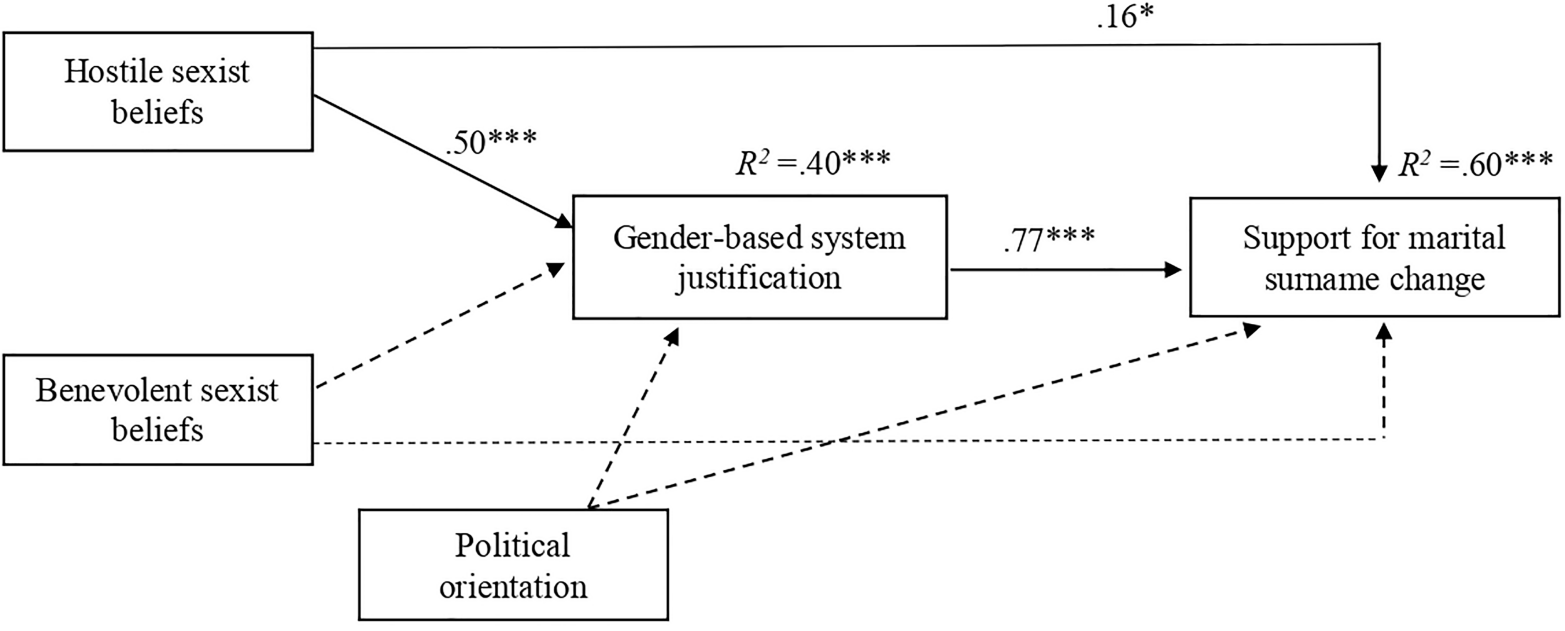
The results of mediation analysis in Study 1. Standardized regression coefficients for the relationship between hostile and benevolent sexism (IVs) and support for marital surname change (DV) as mediated by gender-based system justification and controlled for political orientation, ^***^p<0.001, ^*^p<0.05. Non-significant paths are shown as broken arrows.

We conducted a mediation analysis using PROCESS v.3.0, Model 4 with 5,000 bootstrapped samples (Hayes, 2013) to test whether (1) there is a direct positive significant association between hostile (as compared to benevolent) sexism and support for marital surname change and (2) this association is mediated by gender-based system justification. The percentile bootstrap CI was recommended as least susceptible to the influence of outliers in small samples compared to other popularly used tests (e.g., Creedon and Hayes, 2015). We identified hostile and benevolent sexist beliefs as independent variables, gender-based system justification as the mediator, and support for marital surname change as the dependent variable. In this mediation model, hostile sexism was used as an independent variable, while benevolent sexism was used as a covariate variable. We also controlled for the effects of participants’ political orientation. Results indicated that adherence to hostile sexist beliefs, but not to benevolent sexist beliefs, was a significant predictor of gender-based system justification (see Figure 2). System justification was, in turn, found to positively significantly predict support for marital surname change. Participants’ adherence to hostile sexist beliefs predicted higher support for marital surname change after including gender-based system justification in the model. Results indicated a significant indirect association between adherence to hostile sexism beliefs and support for marital surname change, as mediated by system justification, *b*=0.387, *SE*=0.166, 95% CI [0.06, 0.70]. The total direct effect was significant and large in size (Cumming, 2014), *b*=0.546, *SE*=0.078, 95% CI [0.39, 0.70]. The direct effect of hostile sexism beliefs on support for marital surname change was significant, *b*=0.208, *SE*=0.077, *p*=0.008, 95% CI [0.05, 0.36], when controlled by BS and political orientation. We also conducted a *post hoc* power analysis for indirect effect using the power analysis calculator (see Schoemann et al., 2017). Results yielded sufficient power for the indirect effect of hostile sexism (1.00). The observed indirect effect was large in size (Cumming, 2014). Both direct and indirect effects remained significant after including political orientation in the model as a covariate. Political ideology was not significantly associated with system-justification (*b*=0.074, *SE*=0.056, *p*=0.193) and support for marital surname change (*b*=−0.007, *SE*=0.051, *p*=0.894).

Our first study provided support for the idea that there is a positive association between hostile sexist beliefs and higher support for marital surname among self-identified feminist women in Turkey, mediated by their gender-based system justification. It further suggested that: (i) benevolent sexism did not predict support for marital surname either directly or indirectly through gender-based system justification; (ii) the positive relationship between hostile sexism and support for marital surname change remained significant even when controlling for political orientation. The fact that we observed the aforementioned association among feminist women in Turkey seemingly supports the idea that when existing masculine naming marital conventions are systematically taken by society for granted, they are likely to become endorsed even by feminists, that is, individuals who supposedly stand for more gender equality (Mills, 2003; Stoiko and Strough, 2017). Previous studies (e.g., Stoiko and Strough, 2017) have demonstrated that feminist women had more egalitarian attitudes toward marital naming choice compared to the subsamples of non-feminist women and men. Thus, our study might be the first study to show that there is a link between adherence to hostile sexist beliefs and endorsement of marital surname change mediated by gender-based system justification among women who consider themselves feminist. A better understanding of how different subpopulations of women, including both feminist and non-feminist women, interpret marital naming conventions and their social consequences for gender equality is imperative. In sum, the results of Study 1 were consistent with our prediction that hostile (but not benevolent) sexist beliefs would be particularly related to supporting marital surname change as a gendered practice that reinforces women’s subordination and perpetuates hierarchy in marriage.

## Study 2: Female University Students in Turkey

Study 2 was designed to test our theoretical proposition with a sample of female university students in Turkey. The female student subpopulation has been chosen because, as outlined above, young women in emerging adulthood are likely to be particularly susceptible to established norms and thus tend to endorse societal notions of gender role beliefs that they have construed through the processes of gender socialization (e.g., Nielson et al., 2020).

### Method

#### Participants and Procedure

Data were collected between November 21, 2018 and January 9, 2019. We distributed the link to the survey among university students in a private university in Ankara, Turkey. A sample of 144 undergraduate female students was recruited. Participants were offered course credit for their participation in a research study. They were also provided with non-research alternatives involving a comparable time and effort to obtain the extra credit to minimize the possibility of undue influence (e.g., Beckford and Broome, 2007). Twelve participants withdrew from the participation and thus were excluded from our analysis. A male participant was excluded from the study as this respondent did not match the advertised inclusion criteria (i.e., being a female). The final sample consisted of 131 female university students. Participants’ age ranged from 19 to 41 (*M*=21.05; *SD*=2.06), four participants did not indicate their age. Sensitivity analysis was conducted using G*Power (Faul et al., 2009) showed that this sample size was sufficient for detecting a small effect in a regression analysis (multiple regression: *R*^2^ deviant from zero; power=0.80; *α*=0.05; Cohen’s *f*^2^=0.09).

#### Measures

We used the same scales as those used in Study 1 (a six-item scale for hostile sexism, Cronbach’s *α*=0.82; a two-item scale for benevolent sexism, *r*=0.61, *p*=0.001; a three-item scale for gender-based system justification, Cronbach’s *α*=0.72; a five-item scale for support for marital surname change, Cronbach’s *α*=0.82). Factor analysis showed the same dimensionality of the constructs as in Study 1, with one exception: the item “*Men should be willing to sacrifice their own well-being in order to provide financially for the women in their lives*” was found to cross-load on both hostile sexism and benevolent sexism. As we were interested in replicating Study 1, we treated it as a Hostile Sexism item. The scales and demographic questions, thus, were identical to those used in Study 1.

### Results and Discussion

#### Preliminary Analyses

Means, SDs, and correlations between the variables are presented in Table 2. The mean scores of hostile sexism, gender-based system justification, and the support for marital surname change were rather low. The analysis of descriptive statistics revealed that similar to Study 1, participants scored relatively high on benevolent sexism. Pearson correlation analyses were computed to analyze bivariate associations between the study constructs. Similar to Study 1, greater adherence to benevolent sexism beliefs was associated with greater adherence to hostile sexist beliefs; different from Study 1, greater adherence to hostile sexist beliefs was associated with greater endorsement of gender-based system justification as well as greater support for marital surname change. In contrast, the link between hostile sexism and political orientation was found to be non-significant. Finally, contrary to Study 1, the association between gender-based system justification and support for marital surname change was not significant.

**Table 2:** Means, SDs, and correlations among key variables (Study 2).

Variables	*M (SD)*	1	2	3	4	5
1. Benevolent sexism	3.88 (1.67)	–				
2. Hostile sexism	2.86 (1.24)	0.20^*^	–			
3. Gender-based system justification	2.25 (1.26)	−0.03	0.23^*^	–		
4. Political orientation	3.72 (1.88)	0.14	0.16	0.27^*^	–	
5. Support for marital surname change	2.31 (1.18)	0.16	0.39^***^	0.16	0.24^*^	–

*** *p<0.001*,

* *p<0.05*.

#### Mediation Analyses

**Figure 3:**
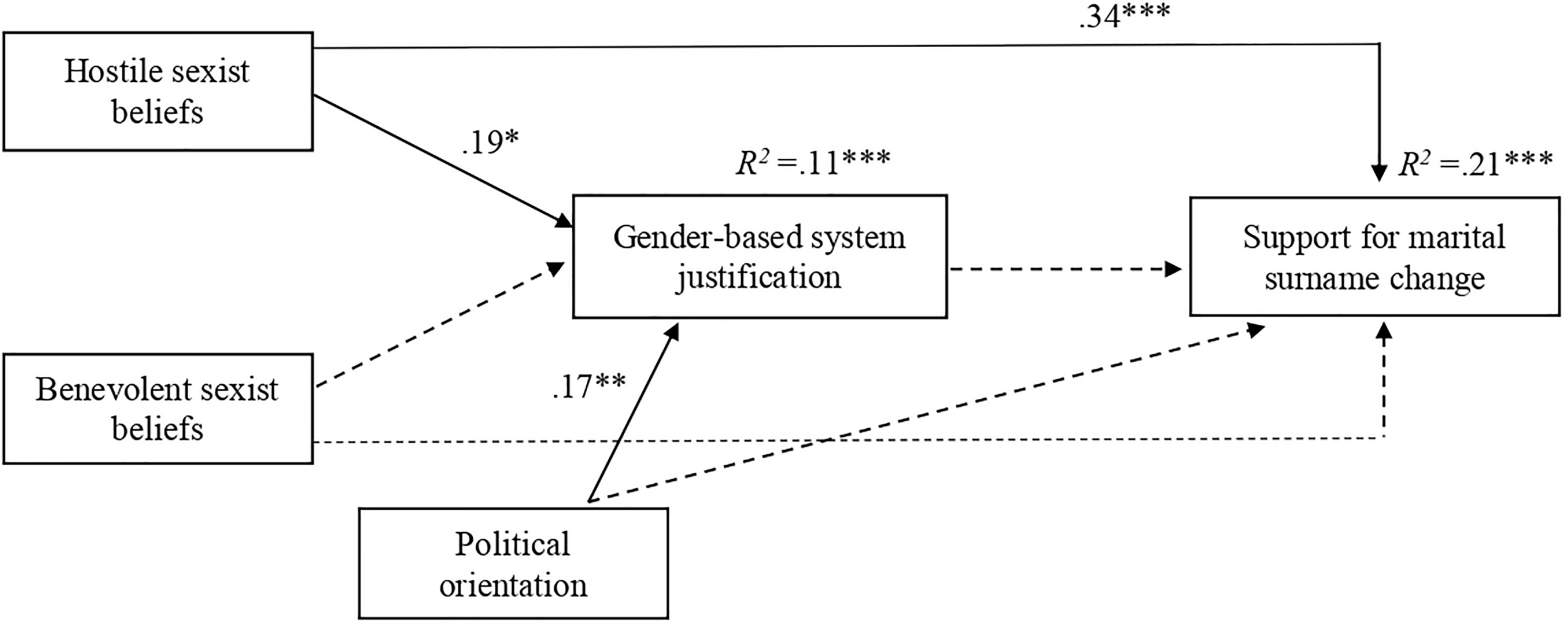
The results of mediation analysis in Study 2. Standardized regression coefficients for the relationship between hostile and benevolent sexism (IVs) and support for marital surname change (DV) as mediated by gender-based system justification and controlled for political orientation, ^***^p<0.001, ^**^p<0.01, ^*^p<0.05. Non-significant paths are shown as broken arrows.

We replicated the same analysis as in Study 1. As expected, the results revealed that adherence to hostile sexist beliefs, but not to benevolent sexist beliefs, was a significant predictor of gender-based system justification (see Figure 3). However, the path from gender-based system justification to support for marital surname change was found to be non-significant, suggesting that the mediation observed in Study 1 did not occur in Study 2. Finally, adherence to hostile sexist beliefs was found to be a significant direct predictor of support for marital surname change, while the direct link between adherence to benevolent sexist beliefs and support for marital surname change was non-significant. The total direct effect was significant and large in size (Cumming, 2014), *b*=0.344, *SE*=0.079, 95% CI [0.19, 0.50]. The significance of the direct association between hostile sexism and support for marital surname change remained unaffected, *b*=0.355, *SE*=0.079, *p*<0.001, 95% CI [0.19, 0.50], after including benevolent sexism and political orientation as the covariates in the model. Political ideology was found to positively significantly predict system-justification (see Figure 3), while it did not predict support for marital surname change (*b*=0.091, *SE*=0.051, *p*=0.078).

In sum, Study 2 conducted among female students in Turkey partially replicated the findings of Study 1 and provided evidence to the idea that hostile (but not benevolent) sexist beliefs predict (i) greater gender-based system justification and (ii) are directly associated with the increased support for marital surname change, while the hypothesized mediation did not occur. The absence of a significant link between gender-based system justification and support for marital surname change could be attributed to a relatively straightforward theoretical model we tested herein. It is possible that these dynamic associations may be more complex, and as such, system justification may manifest among people who make favorable temporal comparisons between their ingroup standing in the past, in the present or in the future (see Caricati and Owuamalam, 2020). Consequently, it would be relevant to replicate the present study by examining whether women’s perceptions of both ingroup upward mobility and the increased political opportunity structure affect the link between gender-based system justification and support for marital surname change, thus leading to the occurrence of a moderated mediation. Further, while Study 2 suggests that young and highly-educated Turkish women are likely to support the traditional practice of changing their marital surname upon marriage to the extent that they adhere to hostile sexist beliefs, it is also important to bear in mind that this gendered practice is legally sustained by the Turkish Civil Code (e.g., Engin and Pals, 2018; Kavas and Thornton, 2019). Some legal scholars have speculated that the government is reluctant to change legal policy in matters of marital surname change such that this gendered practice allows the state to maintain existing gender arrangements in the face of increasing pressures of Western institutions and ideologies (Inal, 2020).

## Study 3: Female University Students in the United States

Study 3 was designed to test our theoretical proposition in the sample of female university students in the United States This country had ranked 53rd among 153 countries in the Global Gender Gap Index 2020 (World Economic Forum, 2020). Compared to Turkey, the United States is considered a WEIRD society and one of the most individualistic cultures in the world, in which people tend to value independence and autonomy (Heine and Buchtel, 2009). In the United States, it is customary for a woman who marries to change her surname to that of her husband. The tradition originated in the law of coverture, which dictated that the identities of a husband and wife merged upon marriage, and that the new unit retained only the husband’s identity (e.g., Kopelman et al., 2009; MacEacheron, 2016). The legal practice was first challenged in the mid-nineteenth century by feminist movements that recognized the oppressive nature of the coverture and its marital naming conventions (Kopelman et al., 2009; MacEacheron, 2016). Starting from 1975 and during the following decade, the procedure allowing a married woman to retain her natal surname became legal in all United States (MacEacheron, 2016). Despite these advances, it is still common for women in the United States to change their birth name upon marriage.

### Method

#### Participants and Procedure

Data were collected between November 27 and December 11, 2018. A sample of 143 undergraduate female students was recruited through the *University of Massachusetts Amherst*, Department of Psychology online participant pool (SONA). Participants were told that they would receive 1 SONA research credit as extra credit for one of their classes. They were also told that participating in this study was not the only way to earn extra credit, and they could contact their professors to learn about other opportunities to earn extra credit. Three participants withdrew from the participation and thus were excluded from our analysis. The final sample consisted of 140 respondents. Participants’ ages ranged from 18 to 27 (*M*=20.16; *SD*=1.37). As in previous studies, sensitivity analysis demonstrated that this sample size was sufficient for detecting a small effect in multiple regression analysis (Cohen’s *f*^2^=0.08).

#### Measures

Similar to Studies 1 and 2, participants were asked to report the extent they agree with the scale items on seven-point response scales (1=*completely disagree*, 7=*completely agree*). We used the same measures as those used in Studies 1 and 2 (a six-item scale for hostile sexism, Cronbach’s *α*=0.84; a two-item scale for benevolent sexism, *r*=0.47, *p*<0.001; a three-item scale for gender-based system justification, Cronbach’s *α*=0.83; a five-item scale for support for marital surname change, Cronbach’s *α*=0.75). Factor analysis showed the same dimensionality of the constructs as in Studies 1 and 2. In particular, results of EFAs revealed that the item “*Men should be willing to sacrifice their own well-being in order to provide financially for the women in their lives*” was again loaded on the HS factor (0.50), as in Study 1. We return to this issue in the General Discussion.

#### Sample Comparisons

To provide a better understanding of the potential cross-cultural (Turkey and the United States) as well as intergroup (self-identified feminist sample and university female student samples) similarities and differences, we performed one-way ANOVAs (see Table 3). We found that the mean level for benevolent sexism across the three studies did not differ to a significant extent. With respect to hostile sexism, *post hoc* comparisons (Tukey’s HSD) indicated that the mean for feminist women in Turkey (Study 1) was significantly lower than for female students in Turkey (Study 2) and female students in the United States (Study 3). With respect to gender-based system justification, *post hoc* comparisons indicated that the mean for feminist women in Turkey was significantly lower than for female students in Turkey. Further, the mean levels for both feminist women in Turkey and female students in Turkey were significantly lower compared to female students in the United States Last, with respect to support for marital surname change, *post hoc* comparisons indicated that the mean level for feminist women in Turkey was significantly lower than for female students in Turkey and female students in the United States.

**Table 3:** Means and SDs among the samples in Studies 1–3.

	Study 1 feminist women in Turkey		Study 2 female students in Turkey		Study 3 female students in the United States		*F*(2,394)
	*M*	*SD*	*M*	*SD*	*M*	*SD*	
Benevolent sexism	3.52^a^	1.84	3.88^a^	1.67	3.94^a^	1.27	2.60
Hostile sexism	1.85^a^	0.75	2.86^b^	1.24	2.59^b,c^	1.07	27.30^***^
Gender-based system justification	1.48^a^	0.88	2.25^b^	1.26	3.04^c^	1.37	54.72^***^
Support for marital surname change	1.43^a^	0.81	2.31^b^	1.18	2.36^b,c^	1.01	46.26^***^

*Means in a row without a common superscript letter differ at the 0.001 level according to Tukey’s HSD test*.

*** *p<0.001*.

### Results and Discussion

#### Preliminary Analyses

Means, SDs, and correlations between the variables are presented in Table 4. The mean scores of hostile sexism, gender-based system justification, and the support for marital surname change were rather low. The analysis of descriptive statistics revealed that participants scored relatively high on benevolent sexism. Pearson correlation analyses revealed that greater adherence to benevolent sexist beliefs was associated with greater adherence to hostile sexist beliefs, greater endorsement of gender-based system justification, right-leaning political ideology as well as greater support for marital surname change. Similar to the findings of Studies 1 and 2, adherence to hostile sexist beliefs was correlated with greater gender-based system justification as well as greater support for marital surname change. As in Study 1, greater gender-based system justification was significantly associated with greater support for marital surname change.

**Table 4:** Means, SDs, and correlations among key variables (Study 3).

Variables	*M (SD)*	1	2	3	4	5
1. Benevolent sexism	3.94 (1.27)	–				
2. Hostile sexism	2.59 (1.07)	0.35^***^	–			
3. Gender-based system justification	3.03 (1.37)	0.21^**^	0.41^***^	–		
4. Political orientation	4.05 (2.01)	0.19^*^	0.36^***^	0.29^***^	–	
5. Support for marital surname change	2.34 (1.01)	0.33^***^	0.41^***^	0.39^***^	0.25^**^	–

*** *p<0.001*,

** *p<0.01*,

* *p<0.05*.

#### Mediation Analyses

**Figure 4:**
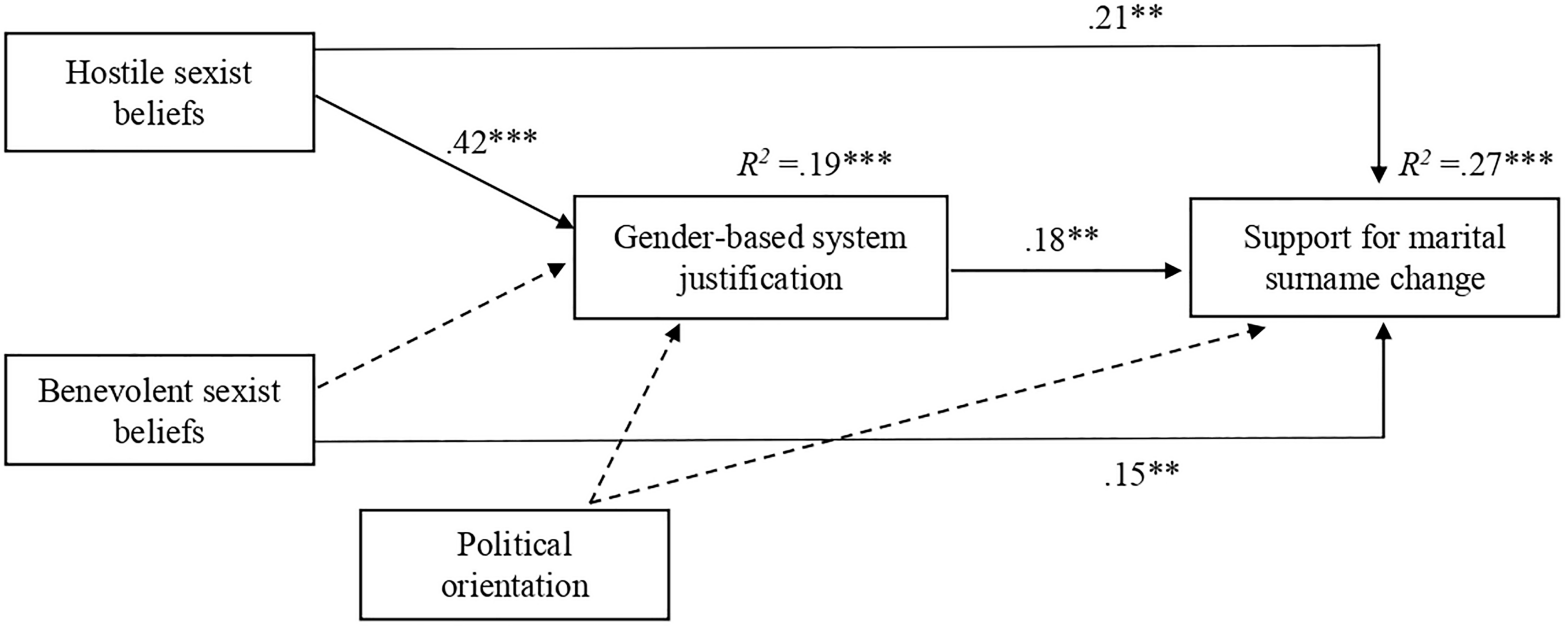
The results of mediation analysis in Study 3. Standardized regression coefficients for the relationship between hostile and benevolent sexism (IVs) and support for marital surname change (DV) as mediated by gender-based system justification and controlled for political orientation, ^***^p<0.001, ^**^p<0.01. Non-significant paths are shown as broken arrows.

The same analyses as in Studies 1 and 2 were carried out. The results indicated that adherence to hostile sexist beliefs, but not to benevolent sexist beliefs, was a significant predictor of gender-based system justification (see Figure 4). Gender-based system justification was, in turn, found to significantly predict higher support for marital surname change. Consistent with our hypothesis, we found a significant indirect association between adherence to hostile sexism beliefs and higher support for marital surname change, as mediated by gender-based system justification, *b*=0.075, *SE*=0.039, 95% CI [0.01, 0.16]. While the direct path from hostile sexism to support for marital surname change was significant as in Studies 1 and 2, in Study 3, we also found respondents’ adherence to benevolent sexism significantly predict support for male-dominated naming practice after marriage. The total direct effect was significant and large in size (Cumming, 2014), *b*=0.285, *SE*=0.082, 95% CI [0.12, 0.45]. The direct effect of hostile sexism beliefs on support for marital surname change was significant, *b*=0.210, *SE*=0.083, *p*=0.013, 95% CI [0.05, 0.38], when controlled by BS and political orientation. The significance of indirect effects remained unaffected after controlling for participants’ political orientation. As can be seen in Figure 4, political ideology was not significantly associated with system-justification (*b*=0.109, *SE*=0.057, *p*=0.056) nor with the support for marital surname change (*b*=0.034, *SE*=0.041, *p*=0.404).

Taken together, the results from Study 3 conducted with the female students in the United States revealed that women’s adherence to hostile sexist beliefs was associated with their greater support for marital surname change through gendered-based system justification. Therefore, these findings fully replicated those of Study 1 (i.e., the direct and indirect associations) and partially replicated those of Study 2 (i.e., the direct association). They also showed that, in the context of the United States, benevolent sexism was directly associated with the support for marital surname change, whereas in our studies conducted in Turkey, this link was non-significant. This result may suggest that in North American culture, not merely hostile sexism but also benevolent sexism may be symbolically driving young women to endorse the marriage norms that perpetuate male dominance in a heterosexual family (e.g., Chen et al., 2009; Scheuble et al., 2012; Stoiko and Strough, 2017). However, importantly and consistent with our hypotheses, hostile sexism was related to the support for marital surname change also indirectly to the extent that young women were likely to justify the existing gender-based system.

## General Discussion

The current research aimed to show that women’s support for marital surname change, an entrenched and prevailing feature of heterosexual marriage in many cultures worldwide, can be understood as a powerful signifier of a strong linear association between the adherence to hostile sexism and system-justifying beliefs. Building on the ideas of the Ambivalent Sexism Theory and the System Justification Theory, we proposed that women’s adherence to both forms of sexist beliefs – hostile sexism and benevolent sexism – would predict their higher tendency to justify existing gender relations in society, which in turn, would positively predict their support for marital surname change. We further hypothesized that hostile (as compared to benevolent) sexism could act as a particularly strong direct predictor of the support for the traditional (husband-centered) naming practice among women, *above and beyond* benevolent sexism, as the former is the ideology that governs resentful preservation of male domination and female subordination in marriage and family. We tested our theoretical model across three cross-sectional studies conducted among feminists in Turkey (Study 1) as well as female students in Turkey (Study 2) and the United States (Study 3). Consistent with our predictions, in all three studies, hostile (but not benevolent) sexism was associated with higher support for marital surname change directly, and in Studies 1 and 3 also indirectly through gender-based system justification. In contrary to our predictions, in Study 2, the link between system justification and support for marital surname change was not significant, and thus the mediation did not occur. Finally, in Study 3 conducted with female students in the United States, benevolent sexism was found to be a complementary direct predictor of support for marital surname change.

With respect to cross-cultural and between-group differences, our analysis revealed that participants in all three studies did not differ in their mean levels of benevolent sexism. Instead, feminist women in Turkey displayed a significantly lower mean level of hostile sexism compared to female students in both Turkey and the United States Some cross-cultural differences were observed with respect to gender-based system justification. In particular, Women in Turkey (Studies 1–2) displayed lower levels of system justification compared to women in the United States (Study 3). Finally, between-group differences were found between feminists and female students as the former group reported significantly lower support for marital surname change. Notwithstanding these observed differences in the mean levels, our findings in general supported the idea that women’s support for male-oriented naming practices is related to their adherence to dominant ideologies about gender and marriage.

Two main messages emerge from the current research. First, our research compellingly demonstrates that hostile sexism, the ideology that resentfully preserves male-dominated gender relations, was significantly associated with the support for male-centerd marital surname change across all studies. Importantly, the observed direct link between hostile sexism and support for marital surname change was positive, linear, robust, and replicable among female students in Turkey and the United States, but also among women who categorized themselves as feminists in Turkey. These findings are consistent with other lines of research suggesting that hostile sexism, the ideology that reinforces idealized notions of traditional male-dominated gendered division, is likely to motivate individuals to engage in behaviors aimed to legitimize female subordination in different life domains, including a heterosexual marriage (e.g., Chen et al., 2009; Day et al., 2011; Petterson and Sutton, 2018; Szastok et al., 2019). Importantly, our analysis is among the first to show how hostile sexism directly predicts support for marital surname change even among the subpopulations of women that might be considered as the frontrunners of social change in society, that is, feminists and female college students. Thus, as long as women endorse this ideology, the more likely they are to be contributing to the legitimization of hegemonic masculinity in a heterosexual marriage by supporting male-dominated naming practices.

Second, consistent with our prediction, we found that in two out of three studies, the indirect association between hostile sexism and support for marital surname change was mediated by gender-based system justification. These indirect links were observed among female students in the United States, an individualistic society in the West that claims progressive gender standards (e.g., Pessin, 2018) and also among feminist women in Turkey, the country that has started to claim more gender equality in marriage over the past decade (see Inal, 2020). However, we also highlight that the full mediation did not occur in Study 2 as there was no significant link between gender-based system justification and support for marital surname among female students in Turkey. The absence of a non-significant link can be explained by the fact that legal norms in Turkey require a married woman to adopt her husband’s last name upon marriage. Although there have been some high-profile cases in this country when women filed lawsuits to the national and international courts to demand a legal possibility to retain their family surname upon marriage, these cases constitute the exception rather than the rule (e.g., see Inal, 2020; Kartal, 2020). It is possible that support for marital surname change among female students in Turkey can be explained by other context-related factors, beyond ideology, such as women’s desire to avoid legal repercussions associated with either retained or hyphenated premarital surname, their fear of costly legal processes, or potential conflicts with their spouses. Therefore, future research in this context should expand on this study to investigate how an array of possibly interwoven processes – group perspectives, group identities, group interests, perceived societal demands, as well as personal motivations – affect women’s support for marital surname change in Turkey, the country that is yet to make women free to decide which surname to use upon marriage (e.g., Uluğ, 2015). Scholars should also examine the extent to which women in Turkey perceive the current Turkish Civil Code to be egalitarian and gender-balanced as well as endorse the need to implement legal reforms to grant women with more rights with respect to their surname retention upon marriage.

Furthermore, it is worth noting that, against our hypothesis, we observed no significant direct and indirect associations between benevolent sexism and support for marital surname change *via* gender-based system justification. In fact, the observed associations may be at odds with some previous theorizing and experimental research in social psychology (e.g., Glick and Fiske, 2001; Barreto and Ellemers, 2005; Barreto et al., 2010; Brownhalls et al., 2021). One explanation for this emergent finding can be that across all studies, the role of a more pacifying and inoffensive benevolent sexism might have been suppressed by power-based hostile sexism as they were examined simultaneously. Future experimental research may scrutinize our theoretical model by systematically manipulating and isolating the independent variables. Further, against our prediction, in Study 3, the direct link between benevolent sexism and support for marital surname change was found to be significant, albeit small in size (Cumming, 2014). It is possible that in the United States, the country where women’s right to retain their maiden name upon marriage has not been disputed in a legal domain since 1975, one’s support for the traditional male-centred naming practice can be the expression of both power-based hostile sexism as well as affectively positive but condescending attitudes to women who embrace traditional gender roles (i.e., benevolent sexism). It is thus plausible that existing legal restrictions, objective gender inequality indexes, normative differences in individualistic and collectivistic cultures, that remained beyond the scope of the current research, can potentially explain the observed discrepancies in the findings. In specific, this observed non-significant association between system justification and support for marital surname change indicate that in a context, where retaining maiden name is prohibited by law, women’s support for marital surname change could be perceived as a clear sign of their ideological beliefs about male supremacy and female subordination (especially among feminist and young women), and thus, predicted by hostile rather than benevolent sexism. However, in a context where changing or retaining maiden surname is a matter of choice, women’s support for this conventional naming practice might not be seen as a mere sign of their adherence to hostile sexism. Prevailing support for this practice can also reasonably manifest women’s tendency to endorse the shared beliefs that men should protect, cherish, and provide for women, especially in marriage. In fact, as previous qualitative research conducted with young women in Western cultures has shown, changing their surnames upon marriage is not just the choice dictated by their approval of traditional norms but also a public declaration of their desire to establish a legally and socially sanctioned union in which two people become one, and this oneness is manifested through a shared family (e.g., Scheuble et al., 2012; Stoiko and Strough, 2017).

Another intriguing issue that our analysis points to is that young women’s ideas about what hostile and benevolent sexism mean in the 21st century may differ from those of their mothers and older generations of women. Quite remarkably, one item that was traditionally proposed to measure benevolent sexism (i.e., “*Men should be willing to sacrifice their own well-being in order to provide financially for the women in their lives*”) was found to be systematically loaded on the hostile sexism sub-scale in the current research among feminist women in Turkey (Study 1) and female students in the United States (Study 3). However, the same item was found to have significant factor loadings onto both hostile and benevolent sexism sub-scales for female students in Turkey (Study 2). One reason behind these observed results can be the fact that many progressive women such as feminists, especially in the WEIRD societies, have become increasingly concerned with their access to full and equal participation in the paid workforce, including their rights to rewards, resources, and opportunities along with men (e.g., Scarborough et al., 2019; England et al., 2020). Besides, according to the official records, in the past year only, women in the United States and women in Turkey earned around 84% of what men earned for the same job (e.g., International Labour Organization, 2020; Barroso and Brown, 2021). It is possible that the notion that “*men should be willing to sacrifice their own well-being in order to provide financially for the women in their lives*” might be interpreted rather as discriminatory, from a progressive point of view, and thus align more neatly with hostile rather than benevolent sexist ideology, as our EFAs revealed. We believe that future qualitative research should examine this idea to better understand what men’s financial provision means for women and what men’s willingness to sacrifice in heterosexual relationships can also entail.

Taken together, the current research contributes to growing evidence that shows that sexist ideology, and in particular, hostile sexism, may be responsible for installing in individuals an antiquated conception of gender relations defined through male domination and female subordination. We acknowledge, however, that our findings should be interpreted as culturally specific. It is possible that in United States and Turkey, women’s adherence to hostile sexism may explain their support for male-oriented naming conventions, given that in both countries, there are still relatively low numbers of women who retain their original surnames even *after* the legislative changes that allow this option. However, as we mentioned at the onset of this paper, different countries vary in their legal arrangements regarding marital naming practices. For example, in Italy, Greece, and Iceland, women keep their original surnames after marriage, whereas in other countries like Japan, women are required by the law to change their surnames upon marriage unless they marry somebody from another country (e.g., Taniguchi and Kaufman, 2020). It is, therefore, important to emphasize that women’s willingness to adopt their husbands’ surnames after marriage should not be considered a direct or a mere indicator of their ideological beliefs about gender hierarchy.

While our research aimed to provide an understanding of the ideological factors behind support for marital surname change among different subpopulations of women in Turkey and the United States, it is also plausible that there are other crucial psychological, lifestyle-related, and socio-demographic factors (e.g., marital status, previous romantic/marriage experiences, and socio-economic status) at play that can either facilitate the endorsement of inegalitarian naming practices or inhibit it. For instance, our research makes an extremely interesting case for further analysis of the role of self-objectification, patriarchal beliefs, perceived stability of male dominance, and perceived legitimacy of gender hierarchy, all of which can help to better understand the obstacles to birth surname retention upon marriage. From a broader cultural perspective, many societies have different naming conventions that may or may not convey gender prejudices, regardless of women’s (or men’s) marriage surname change. Due to the patrilineal surnaming traditions that are still prevalent in many societies (including the societies we studied), women who choose not to change their surname upon marriage can also be seen as internalizing unequal gender relations (since they retained the surname inherited from their father, but not from their mother). They might also not be familiar with the relevant laws concerning the marital naming practices in their country. This might explain why, according to the results of Study 1, even some feminists did not totally object to women’s surname change upon marriage. Several other points of interest can potentially emerge from this line of research with respect to the links between social identity aspirations and gender-based system justification, which have been equivocal so far (see, e.g., Owuamalam et al., 2017, 2021; Pilcher, 2017). In particular, our findings with respect to feminist women in Turkey (Study 1) raise several theoretical questions as to whether social identification with core feminist ideas as well as group-based considerations such as hope to attain equality with the historically advantaged outgroup (Owuamalam et al., 2021) can interact with ambivalent sexism to reduce its effect on the support for marital surname change in this subpopulation of women. Future qualitative research can also shed light on the meaning-making processes to better understand whether feminists in traditionally patriarchal societies such as Turkey consider marital surname change as a gendered practice that reinforces women’s subordination and perpetuates hierarchy in marriage. Besides, future research conducted in non-WEIRD societies such as Turkey should also examine the extent to which support for marital surname change among young women is contingent on the normative content of collective identity (e.g., Turkish women), religiosity (e.g., Muslim or Orthodox) as well as perceptions of national identity threat in the face of ongoing cultural and political processes of Westernization.

Although, we obtained consistent support for our theoretical model in the three studies conducted with different subpopulations of women in Turkey and the United States, this strength should not prevent us from seeing some limitations in our research. First, the studies used a cross-sectional design. Our ability to infer causality or assess the prevalence of phenomena from such a design is limited. Additional limitations of the current research include the use of small convenience samples; therefore, the results should be interpreted with caution due to these limitations. Future studies on marital surname change should be strengthened by the inclusion of nationally representative samples of the adult populations in WEIRD and non-WEIRD societies in order to examine the impact of socio-demographic factors (e.g., age, education, urban–rural residence, socio-economic status, education, and mother’s surname choice upon marriage) as well as social-psychological variables (e.g., ingroup identity, patriarchal beliefs, and gender-egalitarian beliefs) on the link between ambivalent sexism and support for marital surname change. Finally, future research should also expand on the multidimensionality of benevolent sexism and examine under what conditions the underlying factors of benevolent sexism (i.e., complementary gender differentiation, protective paternalism, and heterosexual intimacy), can facilitate women’s agentic behavior, such as the retention of a maiden surname upon marriage. These limitations notwithstanding, the current research is important as it sheds light on the ideological underpinnings of women’s surname choices, thus underscoring the importance of detangling the individual-level processes through which macro-level gender status quo and traditional gender roles are installed.

To conclude, at this point in history, marked by the need for accelerated changes in gender practices and social standards toward more equality, we show that as long women themselves endorse sexist ideologies, the more they are inclined to support gendered practices that are likely to perpetuate their inferior status in gender hierarchy. To break this seemingly vicious cycle, we recommend implementing the interventions at the different stages of education and gender socialization that emphasize the value of egalitarianism among the young generation and advocate for legal reforms that secure more equality between women and men in interpersonal relations, family, and work.

## Data Availability Statement

The raw anonymized datasets are publicly available on the Open Society Framework (OSF): https://osf.io/xka8g/?view_only=258be4fb6b994493b0731bf0e236e5c0.

## Ethics Statement

The studies involving human participants were reviewed and approved by University of Massachusetts Amherst. The patients/participants provided their written informed consent to participate in this study.

## Author Contributions

MC: conceptualization, data curation, formal analysis, investigation, methodology, project administration, and writing – original draft. ÖU: conceptualization, data curation, formal analysis, investigation, methodology, data collection, and writing – review and editing. NS: conceptualization, supervision, and writing – review and editing. BK: data collection and data curation. BÇ: data collection and data curation. All authors contributed to the article and approved the submitted version.

## Conflict of Interest

The authors declare that the research was conducted in the absence of any commercial or financial relationships that could be construed as a potential conflict of interest.

## Publisher’s Note

All claims expressed in this article are solely those of the authors and do not necessarily represent those of their affiliated organizations, or those of the publisher, the editors and the reviewers. Any product that may be evaluated in this article, or claim that may be made by its manufacturer, is not guaranteed or endorsed by the publisher.

## Footnotes

^1^Notable exceptions include Greece, Italy, and Iceland where the legal procedure requires women to retain their birth names when they marry.

^2^Even though we use the well-established terms, WEIRD and non-WEIRD to provide some general characteristics of the two cultural contexts, we note that our samples are highly educated, and thus, tend not to differ on the *E* (educated) dimension. Please see the participants’ characteristics in the three studies below.

^3^The survey also included a few measures of collective action intentions and open–ended questions regarding marital surname change for exploratory purposes.

^4^The original scale consisted of 22 items measuring hostile (HS) and benevolent sexism (BS), 11 items for each sub-scale (Glick and Fiske, 1996). While previous studies generally supported the one-dimensional psychometric structure of HS, the items pertaining to BS’s three theoretical sub-factors (i.e., complementary gender differentiation, protective paternalism, and heterosexual intimacy) were shown to vary across cultures (e.g., GarcÃ­a-SÃ¡nchez et al., 2019). Some recent studies have proposed the shortened scales of HS and BS, showing that the two factors were positively intercorrelated and exhibited the same factor structure as the original, longer scales without sacrificing reliability (e.g., Rollero et al., 2014; Hammond et al., 2018). In light of these previous discussions in the literature, we conducted EFAs to verify if the items adapted from the original scale produced a coherent bi-factorial structure. The two items adopted from the original scale to measure BS comprised its Complementary Gender Differentiation sub-factor.

^5^This item was originally proposed to measure BS, and in particular, protective paternalism (Glick and Fiske, 1996). However, the results of EFAs revealed that in the context of Study 1, it was clearly loaded on the HS factor (0.58). We thus treated this item as a part of HS based on the results obtained in our analysis (see Online Supplementary Materials). We return to this methodological issue in the General Discussion.

^6^These items were selected from the original eight-item scale as they loaded on the same component with the highest factor loadings in our pre-test.
